# Nutrition and Health Claims Spectra of Pre-Packaged Foods on Serbian Supermarket Shelves: A Repeated Cross-Sectional Study

**DOI:** 10.3390/nu13082832

**Published:** 2021-08-18

**Authors:** Dragana Davidović, Katarina Paunović, Danica Zarić, Ana Jovanović, Nadja Vasiljević, Dragana Stošović, Milena Tomanić

**Affiliations:** 1 Institute for Hygiene and Medical Ecology, Faculty of Medicine, University of Belgrade, Dr Subotića 8, 11000 Belgrade, Serbia; katarina.paunovic@med.bg.ac.rs (K.P.); ana.s.jovanovic@med.bg.ac.rs (A.J.); nadja.vasiljevic@med.bg.ac.rs (N.V.); milena.tomanic@med.bg.ac.rs (M.T.); 2 Innovation Centre of Faculty of Technology and Metallurgy, University of Belgrade, Karnegijeva 4, 11000 Belgrade, Serbia; danica.zaric@ihis-nutricionizam.rs; 3 Centre for Hygiene and Human Ecology, Institute of Public Health of Serbia “Dr Milan Jovanovic Batut”, Dr Subotića 5, 11000 Belgrade, Serbia; draganastosovic@yahoo.com

**Keywords:** nutrition claims, health claims, pre-packaged food, food labelling, food policy

## Abstract

Nutrition and health claims (NHCs) are a powerful tool that influence consumers’ final decision on the choice of food products. The purposes of this repeated cross-sectional study were to (i) assess the prevalence of pre-packaged food products containing nutrition and health claims among different food categories, (ii) to determine the type of NHCs labelled on the examined food products, and (iii) to evaluate the trend in the use of NHCs in comparison to the 2012 survey. The survey was conducted immediately before the full enforcement of the new national legislation on NHCs in 2020. It comprised 3141 pre-packaged food products from 10 product categories. In total, 21.2% of food products contained any claim (19.4% contained any nutrition claim; 8.2% contained any health claim). In comparison to the 2012 survey, we observed a rising trend in the presence of NHCs; the use of nutrition claims on food products increased three times and the use of health claims increased 1.3 times in the 2020 survey. Bearing in mind that NHCs are a powerful tool guiding consumers’ food purchase decisions, NHCs should be supported by precise legislation and strict surveillance by the public health authorities.

## 1. Introduction

Non-communicable diseases (NCDs) constitute a major public health challenge as they overburden the health system, pose threat to health progress worldwide, and potentially undermine social and economic development throughout the world [1]. An important way to improve health and to control obesity and NCDs is to focus on reducing the associated risk factors. Research shows that health risks related to high body-mass index and high fasting plasma glucose increase continuously. Certain behavioural risks, particularly those related to diet quality and caloric intake add up to the global burden of disease as well [2]. To improve health on a global scale, public health actions and policies are needed to stop or reverse this trend. Providing information about the risks and harms to health is not enough; concrete strategies are, in turn, needed to enable and facilitate healthier lifestyle choices for all [3].

In order to achieve these public health goals one of the strategies in the domain of food labelling is related to well-designed food labels, providing accurate and easy-to-understand information. Food labels promote health by helping consumers learn about energy values and nutrition content of foodstuffs, raise awareness about reasonable portion size, direct people away from less-healthy to more-healthy foods, and guide their food purchasing decisions [4,5]. In turn, dynamic and developed food labelling motivates industrial competition, forcing food producers to change the formulation of their products to comply with the mandatory or voluntary guidelines [6].

Despite its good intentions, food labelling can be problematic from the consumers’ perspective. An average consumer has to cope with a huge number of food products, a continuous launch of new products, and food packages loaded with too much information on a daily basis [7]. Under such conditions, the consumer looks for a key piece of information that singles out healthier foodstuffs from the less healthy ones, which must be clearly presented and instantly available. That is exactly the role of nutrition and health claims (NHCs) that convey a desired message in a concise, straightforward, and comprehensive way [8,9].

However, there is another point to bear in mind. Given the fact that food manufacturers want their products to attract the attention of consumers [10], NHCs are increasingly used as a marketing tool by the food industry [11]. Unfortunately, the use of incorrect nutrition and health claims in promoting foodstuffs and drinks is not uncommon [12]. As a consequence, there is a possibility that consumers may fall into the marketing trap and get misled by messages that are not compliant with the regulation because they are not based on scientific knowledge. That is the reason why food labelling must be regulated by the law, monitored, and continuously improved.

In the European Union, mandatory and voluntary information available on food products is regulated by the law. In particular, the use of nutrition and health claims is standardized by the European Union Regulation (EC) 1924/2006 [13]. The basic principle of nutrition and health claims is not to mislead consumers. Only health claims scientifically validated by The European Food Safety Authority (EFSA) as well as nutrition claims with established thresholds are allowed to be used [13].

In Serbia, the use of nutrition and health claims is specified by the Rulebook on nutrition and health claims labelled on food declaration, adopted in 2018 and fully in line with the EU Regulation (EC) 1924/2006. Food producers were, however, given time to adjust the NHCs on their products by 31 December 2020 at the latest [14]. At the time of this research in 2020, the new regulation was about to be fully implemented in the country.

A similar survey on the prevalence of NHCs in Serbia was conducted in 2012 when the use of NHCs on pre-packaged food products was not regulated by the law [15]. Bearing in mind the forthcoming changes in the legislation, we also expected the changes in the use of NHCs. Rather than focusing mainly on the current situation, and following the suggestion that prevalence studies should be regularly repeated [16,17], we considered it convenient to undertake a new survey at this moment. Apart from estimating the prevalence of NHCs, and evaluating the trend in the use of NHCs from 2012 to 2020, this survey also provides a further analysis of the types of NHCs, nutrients/ingredients being referred to by NHCs, and body systems/functions targeted by health claims, which could make this survey stand out in the current literature.

The objectives of this study were to (i) assess the prevalence of pre-packaged food products containing nutrition and health claims among different food categories, (ii) to determine the type of nutrition and health claims labelled on the examined pre-packaged food products, and (iii) to evaluate the trend in the use of nutrition and health claims on pre-packaged food products in comparison to the 2012 survey [15].

## 2. Materials and Methods

This cross-sectional study was conducted in Belgrade, Serbia, from September to December 2020 in supermarkets of the three food retail chains which advertise themselves as the largest food retail chain in the region, the largest national food retail chain, and the largest discount food retail in the country [18,19,20]. Once the management of the retail chains granted permission for the study, one store within each chain was selected for the research, preferably the one with the greatest assortment of food products.

The design of the study is based on our previous research in 2012, described in detail elsewhere [15]. In short, we looked into the presence and the type of nutrition and health claims labelled on pre-packaged food products from 10 food categories, the same ones as in the 2012 survey. These food groups were selected because they constitute the major part of typical purchases of an average household according to the Serbian household budget survey [21]. These 10 food categories include breakfast cereals, biscuits, bread and toast, milk, yoghurt, cheese, meat products, ready meals, instant soups, and soft drinks, which were covered by the the FoodEx2 food classification system [22]. The description of the food categories is presented in Table 1.

We collected all pre-packaged food products within the selected 10 food groups that were on display for sale on the shelves or in refrigerators at the time of the study. We made no preference as to the country of origin of the products. Food packages of the same foodstuff, but different weight, were considered separate products because their food labels could have differed in the form and content. To avoid duplicating, we deliberately skipped collecting identical food products found in the second and the third store. We photographed all products during the market analysis from all sides of the package. In total, the survey comprised 3141 pre-packaged food products from 785 brands.

The presence and the characteristics of nutrition and health claims were analysed from the obtained pictures of the food products. Nutrition and health claims were defined in accordance with the Regulation (EC) 1924/2006. The assessment included the following aspects of NHCs:

1. The presence of nutrition claim (NC). Nutrition claim was defined as “any claim which states, suggests or implies that a food has particular beneficial nutritional properties due to the energy it (i) provides, (ii) provides at a reduced or increased rate, or (iii) does not provide; and/or the nutrients or other substances it (i) contains, (ii) contains in reduced or increased proportions, or (iii) does not contain” [13];
2. The type of nutrition claims. All nutrition claims were categorized into one of the following two types: (A) a nutrient content claim, which refers to a nutrition claim that describes the level of a nutrient contained in a food or its energy value (e.g., “low energy”, “source of fibre”), or (B) a nutrient comparative claim, which refers to a nutrition claim that compares the composition of the food in question with a range of foods of the same category (e.g., “energy-reduced”, “increased calcium”) [13];
3. The presence of health claim (HC). Health claim was defined as “any claim that states, suggests or implies that a relationship exists between a food category, a food, or one of its constituents and health” [13];
4. The type of health claims. All health claims were categorized into one of the following four types: (A) a general health claim, which by definition refers to general, non-specific benefits of the nutrient or food for overall good health or health-related well-being (e.g., “healthy”, “fit”, “symbol heart”) [13]; (B) a nutrient and other function claim, referring to (i) the role of a nutrient or other substance in growth, development and the functions of the body, (ii) psychological and behavioural functions or (iii) slimming or weight control or a reduction in the sense of hunger or an increase in the sense of satiety or to the reduction of the available energy from the diet (e.g., “Calcium is needed for the maintenance of normal bones”) [13]; (C) a reduction of disease risk claim, which refers to any health claim communicating that the consumption of a food category, a food or one of its constituents significantly reduces a risk factor in the development of a human disease (e.g., “Plant stanol esters have been shown to reduce blood cholesterol. Blood cholesterol is a risk factor in the development of coronary heart disease”) [13], or (D) a children’s development and health claim, which denotes any health claim referring to the development and health of children (e.g., “Vitamin D is needed for the normal growth and development of bone in children”) [13];

Health claims of the type e.g., “(this food) contains calcium, which contribute to normal blood clotting” were considered as one nutrition claim and one health claim.

5. A format of nutrition and health claims—a non-symbolic or symbolic (a symbolic claim is defined as a picture or a combination of picture and text) [16];
6. A list of nutrients (energy and macronutrients, minerals, vitamins) and other ingredients (fibre, probiotics) that are being referred to by the NHCs [16];
7. A list of effects on the human body or body systems suggested by the health claim. This is a detailed presentation of particular diseases (e.g., anaemia, hypertension), health-related functions (e.g., cardiovascular, digestive function, bones and muscle function, function of the skin), or body systems (e.g., nervous, immune system etc.) targeted by the health claims [16,23].

Finally, we compared the proportion of nutrition and health claims recorded on the food products to the proportion of NHCs obtained in the 2012 study to estimate a possible trend in the use of NHCs. Our first survey in 2012 was conducted following the same research design and comprised the same 10 food categories, 712 brands and 2138 pre-packaged food products [15]. Given the same methodology and similar number of surveyed food products, we consider the results of the two surveys be comparable.

Claims such as “natural” and “organic” were not considered as NHCs as they refer to the production process [16]. They were, therefore, not analysed in this survey. Similarly, claims referring to allergen component in food products, such as “allergen free”, “gluten free”, “contains nuts”, as well as claims referring to food additives, such as “no additives”, “no conserving substances”, “no artificial colour added” were not considered NHCs, as they are not regulated by NHCs legislation [13].

Data are presented as counts (proportion, %). Change from baseline 2012 to 2020 is expressed as a proportion (percentage from baseline). Groups are compared using nonparametric (Pearson Chi-square or Fisher’s exact test, depending on assumptions met) test. All *p* values less than 0.05 were considered significant. All data were analysed using SPSS 20.0 (IBM Corp. Released 2011. IBM SPSS Statistics for Windows, Version 20.0. Armonk, NY, USA: IBM Corp.).

## 3. Results

We collected 3141 pre-packaged food products from 10 product categories. In total, 21.2% of food products contained any claim, one in five food products (19.4%) contained nutrition claims, and 1 in 12 products (8.2%) contained health claims (Table 2).

Nutrition claims were most frequently present on the products from the breakfast cereals category (81.3%). Almost one-third of the products in the bread and toast and soft drinks category had nutrition claims. About one in five products in the biscuits (18.9%), and 14.6% products in the milk category had nutrition claims; in the other four groups, however, nutrition claims were less often reported (around or less than 10% of products) (Table 2).

Health claims were most often present on the products of the following three product categories: breakfast cereals (39.9%), milk (17.7%), and yoghurt (14.8%). Furthermore, they were reported in 1 in 10 food products from the bread and toast category (10.6%), and biscuits category (8.6%). Among the other five categories, health claims were rarely observed (less than 5% of products) (Table 2).

A single food product typically contained 2.4 nutrition claims and/or 1.6 health claims (Table 2). Food products with the highest number of nutrition claims were from the categories of biscuits and soft drinks, containing up to nine nutrition claims per product. Food products with the highest number of health claims were from the yoghurt category and contained up to 11 health claims per product.

The types of nutrition and health claims are presented in Table 3. Overall, 1438 nutrition claims and 427 health claims were recorded on the examined pre-packaged food products.

As for the type, nutrition claims were predominantly categorized as nutrient content claims (e.g., “source of fibre”) (96.8%) (Table 3). Nutrient comparative claims (e.g., “reduced energy value”) accounted for only 3.2% of all nutrition claims and were recorded in the following product categories: soft drinks (26 claims), breakfast cereals (16 claims), yoghurt (2 claims), and meat products (2 claims) (Table 3). They referred to reduced energy value (22 claims), reduced sugar content (20 claims), and reduced fat content (4 claims) of the examined food product. Nutrition claims were always presented as non-symbolic claims.

In total, 60% of all observed health claims were categorized as general health claims (e.g., “healthy for you”, “fit”, “care for health”) (Table 3). Claims related to the role of a particular nutrient in the growth, development and functioning of the body, the so-called nutrient function claims (e.g., “B vitamins contribute to the maintenance of normal energy metabolism”) were observed somewhat less frequently (37.4%). Nutrient function claims were dominant only in the soft drinks (98.4%). Children’s development and health claims were less frequently reported—eight such claims in the breakfast cereals product category, and only one claim in the yoghurt category (“B vitamins contribute to the development of children’s brain”). Finally, only two health claims found on the food products were categorized as reduction of disease risk claims. They were recorded on the products from the milk category and suggested the importance of the “vitamin complex and calcium in the prevention of anaemia”. As for the format of health claims, 348 were presented as non-symbolic claims (81.5%) and 79 as symbolic (18.5%). The most commonly observed symbol was the heart-shaped symbol (on 63 products); it was present on breakfast cereals and some meat products (i.e., canned fish). Three yoghurt and milk products contained a picture of a shield surrounded by microbes, accompanied by the word “immune”. Other pictures found on some yoghurt products were a figure of a strong person lifting weights (3 products) or a slender female silhouette (7 products), typically accompanied with the words “slim” and “fit”.

**Figure 1:**
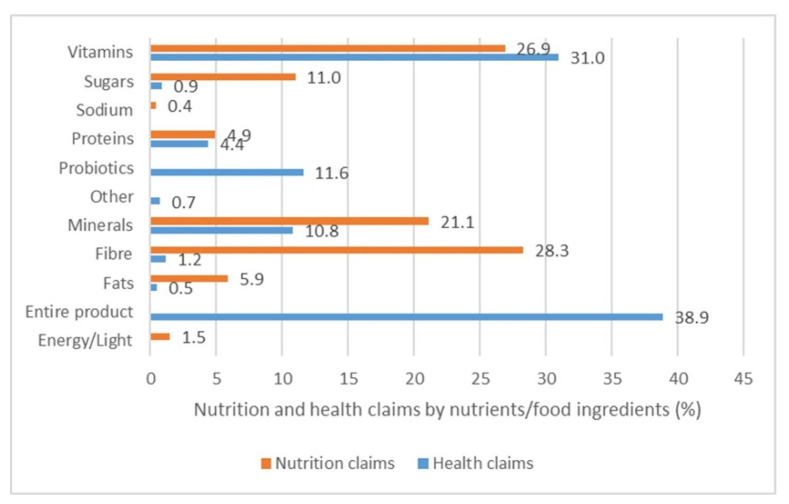
Proportion of nutrition and health claims for each nutrient/ingredient.

Figure 1 presents the nutrients and food ingredients which are being referred to by nutrition and health claims. In general, almost half of all nutrition claims were related to a single vitamin or mineral. In other cases, nutrition claims were linked to fibres (28.3%), sugars (11%), fats (5.9%), and proteins (4.9%) in food products. Nutrition claims referring to energy value, including the claim “light” were recorded on 22 products (1.5% of all NCs). Only five nutrition claims were related to the content of salt/sodium in food products (0.4% of all NCs).

A difference in the nutrient or food ingredient as the object of the claim was observed between product categories. For example, 22.1% nutrition claims found on breakfast cereals, 18.2% on the biscuits, 22.8% on the bread and toast, and 38.9% in the instant soups group referred to their fibre content (e.g., “source of fibre”) predominantly. Among soft drinks products, the most common claims were about vitamins (56.1%), especially vitamins C and E (e.g., “source of vitamin C”), and sugars (15.4%) (e.g., “no sugar”). Furthermore, in the milk and yoghurt category, nutrition claims referred to calcium (20% and 16.1% respectively) and protein content (17.5% and 17.7% respectively) (e.g., “source of calcium”, “source of proteins”).

On the other hand, the majority of health claims (38.9%) referred to the entire product without specifying any nutrient or food ingredient (e.g., “healthy”, “good for you”, “fit”). Such health claims were most often reported in bread and toast (90.5%), cheese (66.7%), breakfast cereals (38.5%), milk (35.6%) and biscuits (35.3%). One-third of health claims referred to vitamins (31%), especially to vitamin B complex (e.g., “B vitamins contribute to maintaining normal energy metabolism”, “B vitamins contribute to reducing fatigue and exhaustion”, “vitamin B1 contributes to normal heart rate”). Overall, 1 in 10 health claims suggested the health-related function of minerals (nine different minerals and unspecified minerals) (10.8%), and of probiotics (11.6%) (Figure 1).

**Figure 2:**
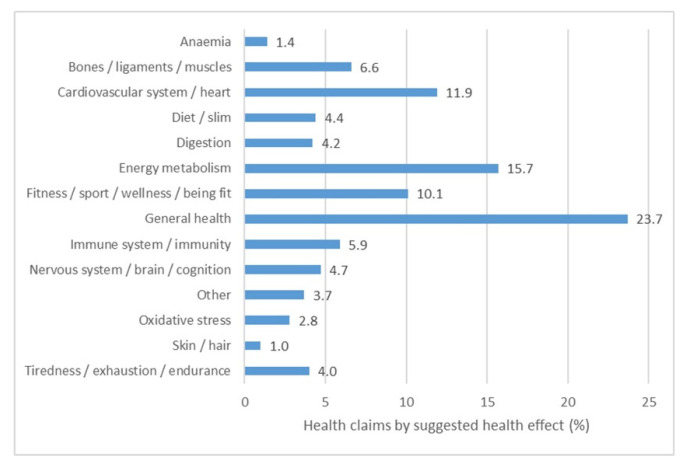
Proportion of health claims according to the suggested health effect on the human body or body systems.

All collected health claims were further categorized according to the specific suggested health effect on the human body or the body systems, which is presented in Figure 2. The largest number of health claims referred to general health (23.7%), and energy metabolism (15.7%). Claims about the beneficial effects of certain nutrients or ingredients on the cardiovascular system and the heart made up for 11.9% of all health claims (e.g., “vitamin B1 contributes to the normal function of the heart”; a symbol of the heart). Furthermore, 10.1% of the claims referred to good physical shape (“beautiful /handsome”), wellness and sports, 6.6% of claims referred to the musculoskeletal system (e.g., “proteins contribute to increasing and maintaining muscle mass”, “proteins contribute to maintaining normal bone condition”), and 5.9% claims referred to the immune system (e.g., “vitamin D /vitamin B6 /vitamin B12 contribute to the maintenance of a normal immune system”, “immune formula”). Health claims less frequently found on food products were related to the brain and the nervous system, digestive system (e.g., “supports better digestion”), reduction of fatigue and exhaustion, weight maintenance and/or weight loss (less than 5% of all health claims each). The “other” claims comprise claims that were recorded on less than 1% of food products each. They referred to various organs ad body functions, including vision, regulation of blood glucose, growth and development, regulation of cholesterol level, water absorption, “elixir”, recovery process, and teeth health (Figure 2).

**Figure 3:**
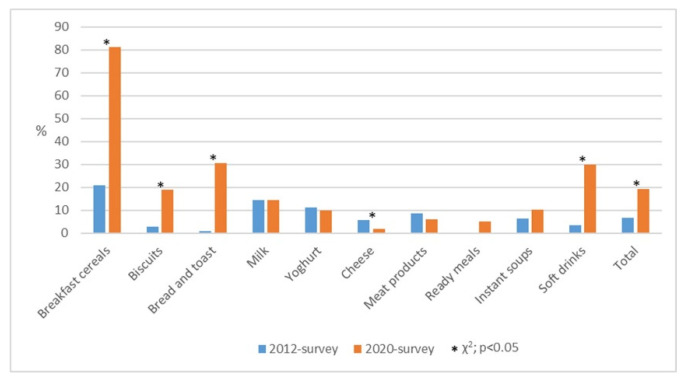
The proportion of pre-packaged food products containing nutrition claims in the 2012 survey [15] and the 2020 survey by product category. % is calculated over the total of products within each food group; * marks statistically significant difference between proportions in the 2012 survey and the 2020 survey, Chi-square test, *p* < 0.05.

The proportion of pre-packaged food products containing nutrition claims in the 2012 survey and the 2020 survey by product category is presented in Figure 3. Overall, the prevalence of nutrition claims increased three times on all examined food products (6.6% in the 2012 survey vs. 19.4% in the 2020 survey; *p* < 0.001).

A significant increase in the proportion of products containing nutrition claims was observed in the following four product categories: bread and toast (38-times higher proportion of nutrition claims in the 2020 survey in comparison to the 2012 survey), soft drinks (nine times increase), biscuits (six times increase), and breakfast cereals (four times increase) (all *p* < 0.001). On the other hand, there was a significant decrease in the proportion of products with nutrition claims in the cheese category (64.3% of reduction; 5.6% in the 2012 survey vs. 2% in the 2020 survey; *p* = 0.02). The proportion of nutrition claims on the examined food products in the other product categories remained stable between 2012 and 2020 (Figure 3).

**Figure 4:**
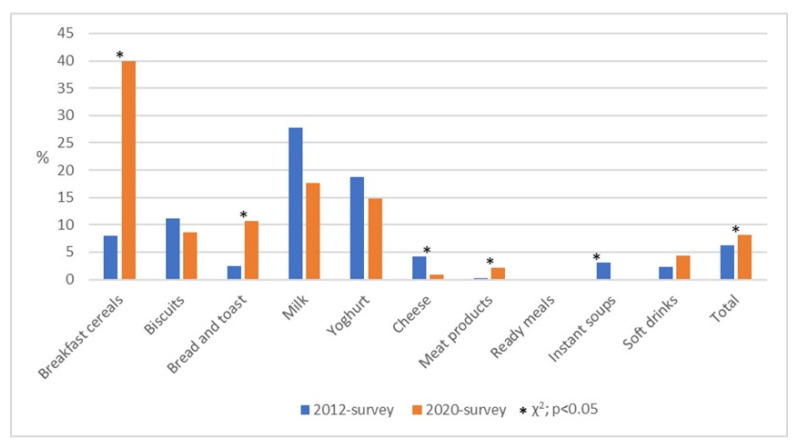
The proportion of pre-packaged food products containing health claims in the 2012 survey [15] and the 2020 survey by product category. % is calculated over the total of products within each food group; * marks statistically significant difference between proportions in the 2012 survey and the 2020 survey, Chi-square test, *p* < 0.05.

The proportion of pre-packaged food products containing health claims in the 2012 survey and the 2020 survey by product category is presented in Figure 4. Overall, the prevalence of health claims increased 1.3 times on all examined food products (6.3% in the 2012 survey vs. 8.2% in the 2020 survey; *p* = 0.011).

The largest increase was observed in the product categories breakfast cereals (*p* < 0.001), and bread and toast (*p* = 0.009), where the proportion of health claims in the 2020 survey in comparison to the 2012 survey was five times higher. A significant increase was also reported in the category of meat products; while almost no meat product contained health claims in the 2012 survey, the proportion came up to 2.2% in the 2020 survey (*p* = 0.006). On the other hand, there was a significant decrease in the proportion of products with health claims in the categories cheese (*p* = 0.012), and instant soups (*p* = 0.04). The proportion of health claims on the examined food products in the other product categories remained stable between 2012 and 2020 (Figure 4).

## 4. Discussion

The presented survey is a second cross-sectional study on nutrition and health claims on pre-packaged foods in Serbia. It follows the design of the first study conducted 8 years prior by repeating data collection in the same food retail chains, from the same product categories, and under the same conditions in which consumers are exposed to food labels in the stores [15]. To the authors’ knowledge, this approach enables the comparability of results and monitoring of a trend in this domain [17].

In short, one in five food products (19.4%) on the market contained nutrition claims in 2020, three times more so than in the 2012 survey [15]. Although the observed increase was significant, the prevalence of nutrition claims was still small in comparison to the other countries. The prevalence of nutrition claims ranged from 30% to 36% in the UK [23], Spain [24], and New Zealand [25], while 47% in Ireland [26]. A study in Australia reported even higher frequency of nutrition claims: in 56% of ultra-processed foods [27].

However, the increase of labelled nutrition claims was not obvious in all examined product categories. The largest increase was recorded in the category of breakfast cereals, which also represent products with the highest proportion of both nutrition and health claims, in line with the findings of other researchers [28,29].

The most commonly recorded nutrition claim on all products, particularly in breakfast cereals, bread and toast, and biscuits was “source of fibre”. Similarly, a study evaluating 3197 food products in the Spanish market reported that “source of fibre” was the most frequently used nutrition claim, which was dominantly present on cereals and biscuits in agreement with our observations [24]. Furthermore, a significant increase in the proportion of labelled nutrition claims was recorded in the category of soft drinks, where the claims were predominantly related to the presence of vitamins and sugars. High prevalence of nutrition claims about vitamins, especially vitamin C, is also found in the five-country study in Europe [16]. Interestingly, in the category of cheeses, nutrition claims were less present than in the 2012 survey, while in the other dairy products, yoghurt and milk, there was no significant change [15]. One of the possible explanations is that the local dairy industry that suggested healing properties of their products was under public pressure to stop advertising such products as sources of nutrients, bioactive compounds, and other substances which are not regulated by the Rulebook [14]. Another noteworthy outcome of the presented study was the finding of only five nutrition claims related to the content of salt/sodium in food products (0.4% of all nutrition claims). However, the result would have been different if we had examined other product categories, such as nuts, seeds, snack food, or baked goods [17]. A repeated cross-sectional analysis of Canadian food products between 2010 and 2013 finds an increase in sodium-related claims from 4.5% to 4.9% [17]. The prevalence of 6.3% of sodium-related claims was reported in a Spanish study [24].

Another area of this research was the presence of health claims, found on one in twelve food products in 2020, slightly more frequently than in the 2012 survey [15]. Nevertheless, this increase is not nearly as big as the increase in the use of nutrition claims. In comparison to some other countries, the presence of health claims on food products on the Serbian market is slightly lower. For example, a survey conducted in five EU countries, the UK, the Netherlands, Germany, Slovenia, and Spain finds that 11% of food products contain health claims [16]. Furthermore, the prevalence of health claims ranged from 13% in another Slovenian work [30], to 15% in another UK study [23], and to 22% in Brazil [31]. The differences in national legislations at the time of the survey, the differences in the examined food categories, and different definitions of nutrition and health claims used in the studies could account for the observed discrepancies across the studies.

The increase in the use of health claims varied by food categories in the presented study. The largest increase in the proportion of products with health claims was in the category of breakfast cereals, and bread and toast. This finding is noticeable as breakfast cereals are generally considered “healthy” for the general population, being a source of complex carbohydrates, micronutrients (vitamins and minerals), and fibre [32]. In the authors’ opinion, the presence of health claims on breakfast cereals is highly questionable and must be strictly monitored because of the often-unhealthy nutritional profile of these food products. A study exploring the association between health claims and the nutritive quality of breakfast cereals found that the presence of health claims did not correlate with the overall product healthiness and did not designate “healthy” foods [33]. An additional evaluation of the nutrition quality of breakfast cereals reported no substantial differences in terms of nutritional profile in breakfast cereals with and without NHCs [34].

According to many researchers, this observation does not only relate to breakfast cereals, but can be generalized to other unhealthy products. A UK study that assessed nutrient profile of different products carrying nutrition and health claims reports that products with NHCs have a slightly more favorable nutrient profile than those without NHCs [23]. As previously mentioned, health claims can be substantiated by a nutritionally high-quality product, but on the other hand, they have the potential to mislead and attract consumers by presenting nutritionally poor foods as healthy. Food products containing NHCs should meet certain nutrition standards, in order to decrease the possibility that producers only promote a particular beneficial nutrient but do not indicate the presence of a less beneficial or potentially harmful nutrient in the same product (e.g., labelling “source of fibre” and hiding fat and salt content) [23,29].

In the categories of cheeses and instant soups, health claims were less frequently prevalent than in the 2012 survey [15]. We believe that the enforcement of the new Regulation forced many manufacturers of these food products to comply with health claims in this domain. For example, we used to record claims about the miraculous healing effect of the examined goat cheeses in the 2012 survey, but we no longer came across such statement in the current study. Similarly, in our culture, chicken soup is considered a homemade remedy for cold, so statements referring to its healing properties used to be present on instant soup packages back in 2012, but not in 2020.

As for the category of meat products, health claims were recorded for the first time in the presented study, which was not the case in the 2012 survey [15].

Furthermore, in this survey, we frequently found general health claims (“healthy”, “fitness”, “good for you”) that do not specify any particular nutrient to which the statement refers. The symbol of the heart is also most often noticed without providing information regarding a nutrient with claimed effect on the cardiovascular system (especially among breakfast cereals). It is interesting to note that in Serbian language the word “healthy” has a double use. On the one hand, the word “healthy” is an adjective related to the noun health, but it can also be used as a greeting, meaning “hello”. This play-on-words can account for the common presence of the statement “healthy” on many domestic food products.

Nutrition and health claims represent the way health-related information communicates with the consumer. The presented study supports the importance of careful reading and good understanding of all data provided on food labels in order to make informed choices. However, we cannot always be sure how consumers understand and interpret health claims. We may assume that consumers do not make a difference between nutrition and health claims the way professionals do, which implies that experts who propose health claims should collaborate with consumers to formulate claims in a comprehensive, relevant and familiar way, yet based on scientific knowledge [9,35]. Many studies maintain that consumers prefer simple, clear messages and not complex scientific terms [9,35,36]. Yet another question is whether consumers believe in the messages conveyed by the health claims. Research finds that the believability of nutrition and health claims depends on consumers’ knowledge, perception of the product, and some characteristics of the product; believability, in turn, influences consumers’ purchasing decisions and consumption of the product [37].

These examples point to the importance of consumers’ knowledge, motivations, and expectations guiding their purchase decisions. Studies show that consumers with higher nutrition knowledge and/or higher health motivation were reported to spend more time looking at the nutrition and health claims, which may or may not influence their purchase decisions [7]. Studies in the experimental setting show that the selection and purchase of food products depend on the visual attention that consumers pay to the product and nutrition and health claims labeled on the product [38], the time consumers spend looking at specific claims, perceived healthiness and tastiness of the product, as well as on the price of the product [7]. Nevertheless, visual representations on food packages may not be important for all consumers; according to a Slovenian study, younger consumers rely on the images, whereas most consumers find nutrition and health claims more convincing [39]. Given the fact that the use of NHCs is constantly increasing, there is a strong need to conduct a similar survey among Serbian consumers on their knowledge, motivation and preferences on NHCs.

The presented survey is not without limitations. First, the analysis was based on 10 product categories available on the market. Although it would be best to analyse all products from all product categories in all stores in one country, this is very difficult to pursue in practice due to limited resources, funds, and time. For this reason, researchers typically target certain product categories; this custom may, however, make it difficult to compare the results across studies if the definition of these product categories varies across studies, cultures, or markets. Second, a cross-sectional design can be considered a limitation of such studies. To overcome this problem, and to monitor changes in food labels over time, it is recommended that the study be repeated at some intervals, e.g., after the introduction of the new regulation [16,17]. For that reason, we applied an identical method, i.e., we visited the same stores and selected the same product categories, which is the main strength of the study. However, we did not match food products in our comparative study, which should be overcome in further analysis.

We believe that similar research needs to be repeated in the future to reveal the positive and negative factors that could have brought about a change in labelling practices in our country.

## 5. Conclusions

This study reveals that the use of nutrition and health claims is growing in Serbia. The imminent change in the Serbian legislation has led to significant changes in the presence of nutrition and health claims from 2012 to 2020.

Bearing in mind the need to prevent non-communicable diseases and the fact that NHCs are a powerful tool that influences the final decision of consumers on the choice of food products, we realize that the use of NHCs requires close attention of public health authorities. Future studies should also focus on the nutritional quality of foods that contain nutrition and health claims.

To achieve this public health goal and point to a healthier product, the correct use of nutrition and health claims should be supported by precise legislation and strict surveillance by the public health authorities. We firmly believe that NHCs must tell “the truth, the whole truth, and nothing but the truth” so that consumers can rely on them at every purchase.

## Figures and Tables

**Table 1:** The description of the food categories included in the 2012 survey and 2020 survey.

Product Category	Description
Breakfast cereals	Cereal products that are consumed for breakfast: muesli, flakes, bran cereals, puffed cereals
Biscuits	Products containing flour, sugar and fat, with or without a filling or a topping: hard, soft and confectionery biscuits
Bread and toast	Products based on flour, containing the word ‘bread’/’toast’ on the packaging
Milk	Milk (cow’s, sheep’s or goat’s) and milk drinks with other ingredients e.g., chocolate, vanilla; milk shakes
Yoghurt	Yoghurt and other fermented milk products, natural or with added fruit and/or sugar
Cheese	Milk product, containing the word ‘cheese’ on the packaging excluding dairy-free cheese
Meat products	Products of animal origin produced from muscle, fat, intestines and skin by different technological methods of processing and preservation: cold cuts, pates, sausages, canned meat, and fish excluding fresh and frozen meat
Ready meals	Ready-packed meals containing protein sources (meat, fish or similar), carbohydrate sources (potatoes, pasta or similar) and vegetables, as well as the variety of products that do not contain all of the three elements
Instant soups	Powdered products which dissolve in water; containing the word ’soup’ on the packaging
Soft drinks	Carbonated, non-carbonated and fruit drinks stored in bottles or cans, and used as refreshments

**Table 2:** Number of collected food products, the number (proportion) of food products containing NHCs, and the average number of NHCs by product category.

Product Category	No. of Collected Food Products	No. (% ^1^) of Products Containing Any Claim	No. (% ^1^) of Products Containing Nutrition Claims	Average No. of Nutrition Claims Per Product ^2^	No. (% ^1^) of Products Containing Health Claims	Average No. of Health Claims Per Product ^3^
Breakfast cereals	203	168 (82.8%)	165 (81.3%)	2.8	81 (39.9%)	1.6
Biscuits	567	107 (18.9%)	107 (18.9%)	2.9	49 (8.6%)	1.4
Bread and toast	189	61 (32.3%)	58 (30.7%)	2.3	20 (10.6%)	1.1
Milk	130	29 (22.3%)	19 (14.6%)	2	23 (17.7%)	2
Yoghurt	290	55 (19.0%)	29 (10.0%)	1.8	43 (14.8%)	1.6
Cheese	351	9 (2.6%)	7 (2.0%)	1.3	3 (0.9%)	1
Meat products	593	45 (7.6%)	35 (5.9%)	1	13 (2.2%)	1.1
Ready meals	96	5 (5.2%)	5 (5.2%)	1	0	0
Instant soups	156	16 (10.3%)	16 (10.3%)	1.1	0	0
Soft drinks	566	172 (30.4%)	169 (29.9%)	2.1	25 (4.4%)	2.6
Total No. of products	3141	667 (21.2%)	610 (19.4%)	2.4	257 (8.2%)	1.6

^1^ % is calculated over the total of products within each food group, ^2^ only food products with nutrition claims were considered for the calculation, ^3^ only food products with health claims were considered for the calculation.

**Table 3:** The types of nutrition and health claims by product category.

Product Category	Nutrition Claims			Health Claims			
	No. of Nutrition Claims	No. of Nutrient Content Claims (%) ^1^	No. of Nutrient Comparative Claims (%) ^1^	No. of Health Claims	No. of General Health Claims (%) ^1^	No. of Nutrient Function Claims (%) ^1^	No. of Reduction of Disease Risk Claims and Children’s Development (%) ^1^
Breakfast cereals	462	446 (96.5%)	16 (3.5%)	130	96 (73.8%)	26 (20.0%)	8 (6.2%)
Biscuits	313	313 (100%)	0	68	43 (63.2%)	25 (36.8%)	0
Bread and toast	136	136 (100%)	0	21	20 (95.2%)	1 (4.8%)	0
Milk	39	37 (94.9%)	2 (5.1%)	46	29 (63.0%)	15 (32.6%)	2 (4.4%)
Yoghurt	62	60 (96.8%)	2 (3.2%)	81	50 (61.7%)	30 (37.0%%)	1 (1.3%)
Cheese	9	9 (100%)	0	3	3 (100%)	0	0
Meat products	35	35 (100%)	0	14	14 (100%)	0	0
Ready meals	5	5 (100%)	0	0	0	0	0
Instant soups	18	18 (100%)	0	0	0	0	0
Soft drinks	359	333 (92.8%)	26 (7.2%)	64	1 (1.6%)	63 (98.4%)	0
Total	1438	1392 (96.8%)	46 (3.2%)	427	256 (60.0%)	160 (37.4%)	11 (2.6%)

^1^ % is calculated over the total number of nutrition/health claims within each food group.
